# Understanding Child Stunting in India: A Comprehensive Analysis of Socio-Economic, Nutritional and Environmental Determinants Using Additive Quantile Regression

**DOI:** 10.1371/journal.pone.0078692

**Published:** 2013-11-04

**Authors:** Nora Fenske, Jacob Burns, Torsten Hothorn, Eva A. Rehfuess

**Affiliations:** 1 Institut für Statistik, Ludwig-Maximilians-Universität München, Munich, Germany; 2 Institut für medizinische Informationsverarbeitung, Biometrie und Epidemiologie, Ludwig-Maximilians-Universität München, Munich, Germany; Aga Khan University, Pakistan

## Abstract

**Background:**

Most attempts to address undernutrition, responsible for one third of global child deaths, have fallen behind expectations. This suggests that the assumptions underlying current modelling and intervention practices should be revisited.

**Objective:**

We undertook a comprehensive analysis of the determinants of child stunting in India, and explored whether the established focus on linear effects of single risks is appropriate.

**Design:**

Using cross-sectional data for children aged 0–24 months from the Indian National Family Health Survey for 2005/2006, we populated an evidence-based diagram of immediate, intermediate and underlying determinants of stunting. We modelled linear, non-linear, spatial and age-varying effects of these determinants using additive quantile regression for four quantiles of the Z-score of standardized height-for-age and logistic regression for stunting and severe stunting.

**Results:**

At least one variable within each of eleven groups of determinants was significantly associated with height-for-age in the 35% Z-score quantile regression. The non-modifiable risk factors child age and sex, and the protective factors household wealth, maternal education and BMI showed the largest effects. Being a twin or multiple birth was associated with dramatically decreased height-for-age. Maternal age, maternal BMI, birth order and number of antenatal visits influenced child stunting in non-linear ways. Findings across the four quantile and two logistic regression models were largely comparable.

**Conclusions:**

Our analysis confirms the multifactorial nature of child stunting. It emphasizes the need to pursue a systems-based approach and to consider non-linear effects, and suggests that differential effects across the height-for-age distribution do not play a major role.

## Introduction

Child undernutrition is the cause of one third of deaths in children under five [1]. It produces serious health, social and economic consequences throughout the life course [2]–[4] as well as across generations [5], making it the leading risk factor among children under five worldwide [6]. Low height-for-age or stunting reflects a failure to reach linear growth potential, and is a key indicator of chronic undernutrition. Globally, depending on the precise definition and estimate used, between 171 million [7], [8] and 314 million [9] children under five are currently classified as stunted, with 90% of this burden occurring in 36 African and Asian countries [1]. Between 1985 and 2011 the prevalence of moderate-to-severe stunting has declined from 47% to 30% [9], but progress has been highly uneven, and stunting rates in the most affected world regions have largely remained static [9], [10].

To date, most of the large-scale programmes to address stunting have fallen behind expectations. Systematic reviews of the effectiveness of some of the major nutrition interventions, such as promotion of breastfeeding [11], promotion of complementary feeding through education or food provision [3], [12]–[14], and supplementation with single or multiple nutrients [15], [16] usually show significant impacts on behaviour but modest and context-dependent impacts on height gain or stunting prevalence [17]. Moreover, few children in the developing world currently benefit from optimal breastfeeding practices, as well as sufficient dietary diversity and meal frequency [7]. In contrast, the history of most industrialized countries suggests that virtually all stunting can be averted, making the failure to make rapid progress all the more disconcerting. Therefore, it is essential to revisit the assumptions that underlie current intervention practices.

It is broadly accepted that child stunting is the outcome of multiple risk factors. Nevertheless, much of the modelling to assess presumed cause-effect relationships in observational epidemiology and effectiveness research tends to reduce this complex interplay of risk factors through focusing on single risks and interventions. The recent emphasis on the relevance of systems approaches in epidemiology [18]–[22] implies, however, that the determinants of stunting must be examined in their entirety, if we do not want to risk incorrect estimates of risk factors and interventions as a result of oversimplifications in modelling approaches. Furthermore, it has been suggested that the impact of risk factors (and interventions) on the lower tail of the distribution might differ considerably from their impact on population means [12]; therefore a careful exploration of such differential effects is merited. Finally, the assumption that many “established” risk factors exert their effect in a linear way is being challenged by emerging evidence of non-linear effects [23].

In light of the above, this study aims to undertake a comprehensive analysis of the determinants of child stunting, and to explore whether the three above-described common-practice simplifications in modelling approaches are appropriate. More specifically, the objectives are to (i) capture the interconnectedness between multiple risk factors through an integrated analysis, (ii) explore whether differential effects emerge across the height-for-age distribution, and (iii) test whether non-linear effects play a role. To do so, we developed a conceptual diagram of potential determinants, and applied the innovative statistical approach of additive quantile regression with boosting estimation to data from the Indian National Family Health Survey (NFHS). With an estimated stunting prevalence of 51% and 61 million stunted children, India is the most affected country in the world [1] and improvements in the last two decades have been almost negligible [24].

## Materials and Methods

### Conceptual diagram and corresponding literature

**Figure 1:**
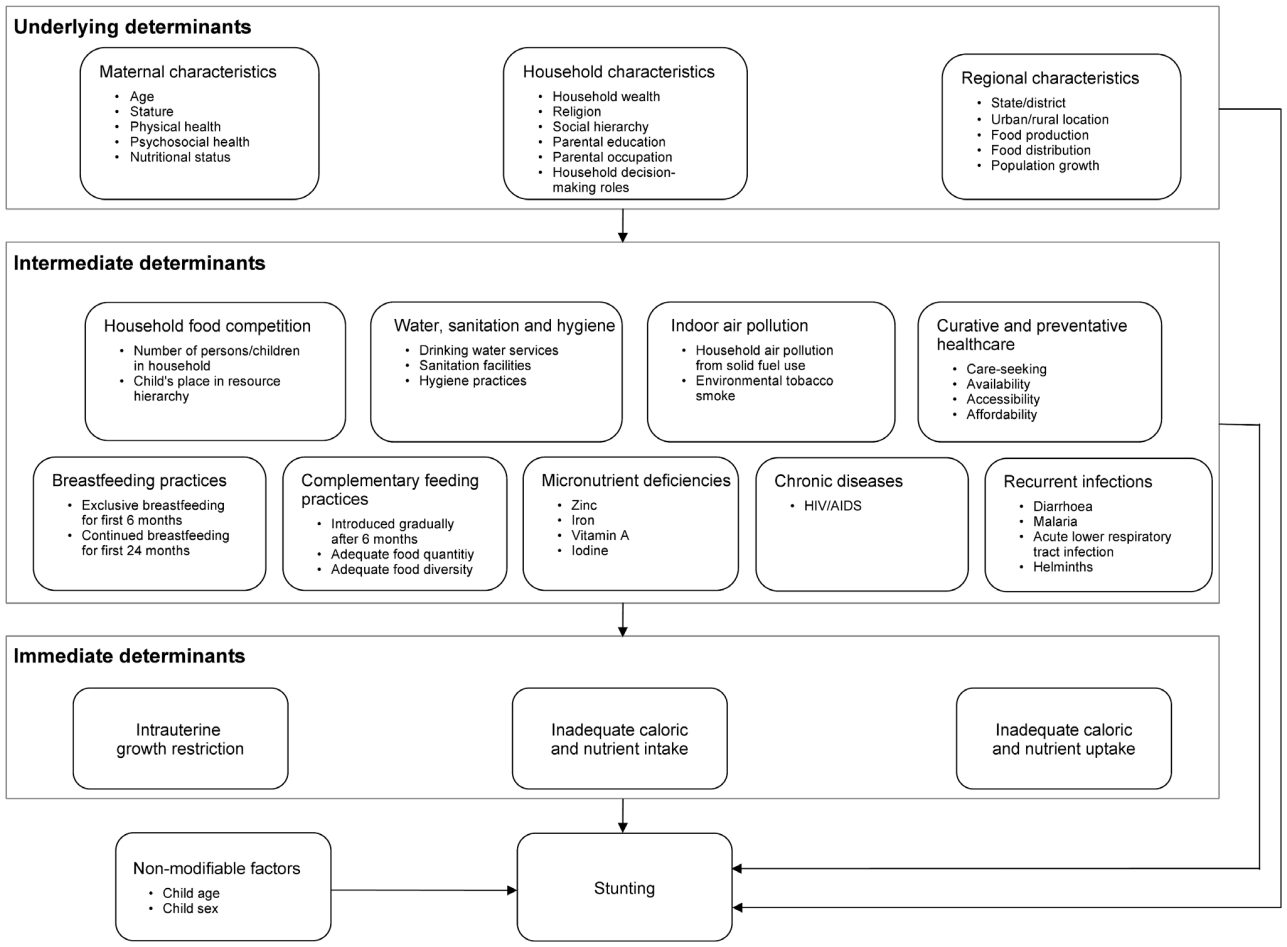
Schematic diagram of the multiple determinants of child stunting, structured by layer (i.e. immediate, intermediate and underlying determinants) and groups of determinants (e.g. maternal characteristics, household food competition, intrauterine growth restriction).

We pursued an evidence-based approach to mapping the complex interplay of factors that determine whether a child becomes stunted or not. Drawing on the well-known UNICEF framework [25], [1] and *a priori* reasoning, we conducted extensive literature searches and structured our findings in a diagram of immediate, intermediate and underlying determinants of child stunting comprising sixteen main groups of determinants (**Figure 1**). In theory, a comprehensive analysis should consider all of these determinants.

Age and sex are critical *non-modifiable factors* [3], [26]. The most important modifiable immediate causes of stunting are *inadequate caloric and nutrient intake* and *uptake* [25]. *Intrauterine growth restriction (IUGR)* is also known to affect long-term growth and development [27]–[29].

Large families and scarce, poorly distributed resources may limit food access and prompt *household food competition*. Various studies have found crowding [30], number of children living in a household [27], birth order [30], and birth interval [31], [32] to be associated with stunting.

While improved *water, sanitation and hygiene practices* protect against stunting [33]–[35], *indoor air pollution* from solid fuel use has been suggested as a risk factor [30], [36], [37]. Environmental tobacco smoke (ETS) shows positive, negative and null associations depending on the country [38].

The World Health Organization (WHO) recommends that infants be exclusively breastfed for six months [39], and that subsequently breastfeeding be continued alongside the gradual introduction of nutritiously diverse and safe solids at an appropriate frequency [40], [24]. Numerous studies have found significant effects of *breastfeeding practices* and *complementary feeding practices* on stunting [41]–[45], [11]–[14].

*Micronutrient deficiencies*, in particular vitamin A [46], iron [47], zinc [16], and iodine [48], may also contribute [25], [49]. It is, however, unclear whether supplementation of single micronutrients is effective in promoting healthy growth, whereas supplementation with multiple micronutrients has shown encouraging results [50], [51], [15].

*Recurrent infections*, such as diarrhea [52], acute respiratory infections [53], and helminthes [54], [55] along with *chronic diseases* such as HIV/AIDS [56], [57], may also increase risk, as these conditions can reduce appetite, hinder uptake of nutrients or increase metabolic requirements and nutrient loss [58].

Availability, accessibility and affordability of appropriate *healthcare* during pregnancy, birth, the postnatal period and continuing into childhood [59], [60] determines a health system's ability to prevent, diagnose and treat chronic undernutrition [61].

*Household characteristics*, measured as wealth [62], [63], [60], religion [27], social hierarchy [64], maternal [64], [65] and paternal education [65], occupation [66], and household decision-making roles [67], [68], are major underlying determinants.

*Maternal characteristics*, such as age [69], [60], stature [70]–[73], [60], nutritional status [74], physical and psychosocial health, also play a role. For example, children born to HIV-infected [75] or depressed mothers [76], [77] are at greater risk of being stunted than children of healthy mothers.

Stunting prevalence varies widely both between [69] and within countries [78]. Relevant *regional characteristics* include urban/rural location and the capacity to produce food (e.g. local climate, land use [79], [66]; and distribute food (e.g. road infrastructure, markets). Population growth, land degradation and increasing climate variability are all predicted to strain food production and increase the burden of child undernutrition [80].

### Data and variables

We used data from the Indian NFHS for the years 2005/2006, a large, well-established, nationally representative survey based on a multi-stage cluster sample design that provides high-quality information on the health and nutrition of women and children [81]. The National Family Health Survey is the Indian equivalent of the Demographic and Health Surveys, a series of standardised surveys which are routinely conducted in more than 70 developing countries. All data are in the public domain and can be downloaded, after registration, from http://www.measuredhs.com. In our analysis we focused on children aged 0–24 months, as stunting prevalence progressively increases until it reaches a plateau at around 24 months [1], [3], [26] and as it becomes very difficult to reverse stunting after this critical time window [82]. Stunting is measured by a Z-score of standardized height-for-age according to the WHO child growth standards [83]; stunted or severely stunted children are those with a Z-score below -2 or -3, respectively [1].

**Figure 1** served as a basis for identifying relevant variables within each group of determinants; all variables, as well as their definitions and empirical distributions in the final dataset are shown in **Table 1**. We carefully investigated all potential variables to populate a determinant from the diagram and chose suitable variables or proxies based on descriptive statistics. The final dataset contains variables to populate most groups, but measures or suitable proxies of IUGR, nutrient intake and uptake, chronic diseases and recurrent infections were not available. For other groups, we could not assess all characteristics of interest, for example in relation to maternal psychosocial health, zinc and ETS. We examined various measures of curative and preventative healthcare, e.g. possession of a health card, health facility visit in past three months, care-seeking for episodes of respiratory infections, or diarrhoea during the two weeks preceding the survey. We ultimately settled for the number of antenatal visits as a proxy for care during pregnancy and childbirth, and constructed a vaccination index based on vaccinations against measles, polio, tuberculosis (BCG) and diphtheria, pertussis and tetanus (DPT) as a proxy for care during childhood.

**Table 1:** Overview of variables and their empirical distributions contained in the final dataset with N = 12 176 observations, arranged by groups of determinants from Figure 1.

Variable	Values/Description	Number	Percentage
			
*Stunting*			
Z-score for height-for-age	Mean: −1.37, Median: −1.44, Sd: 1.79, Range: [−6, 6]		
Child is stunted	No	7699	63.2%
(Z-score≤−2)	Yes	4477	36.8%
Child is severely stunted	No	10089	82.9%
(Z-score≤−3)	Yes	2087	17.1%
			
*Non-modifiable factors*			
Child age [months]	Mean: 12.46, Median: 13, Sd: 6.62, Range: [0, 24]		
Child sex	Male	6317	51.9%
	Female	5859	48.1%
			
*Maternal characteristics*			
Maternal age [years]	Mean: 25.66, Median: 25, Sd: 5.21, Range: [15], [49]		
(at interview)			
Maternal BMI [kg/m^2^]	Mean: 20.10, Median: 19.52, Sd: 3.26, Range: [12.04, 40.34]		
(at interview)			
			
*Household characteristics*			
Household wealth	Poorest	2180	17.9%
(Composite measure of a	Poorer	2226	18.3%
household's living standard	Middle	2463	20.2%
based on ownership of 33	Richer	2726	22.4%
assets; households are	Richest	2581	21.2%
grouped in five quintiles)			
Religion of household head	Hindu	8683	71.3%
	Muslim	1714	14.1%
	Christian	1232	10.1%
	Sikh	224	1.8%
	(Neo-)Buddhist	137	1.1%
	Other	186	1.5%
Caste/tribe of household head	Scheduled caste	2222	18.2%
	Scheduled tribe	2098	17.2%
	Other backward class	4188	34.4%
	None of them	3668	30.1%
Maternal education [years]	Mean: 5.40, Median: 5, Sd: 5.16, Range: [0, 20]		
Partner's education [years]	Mean: 7.21, Median: 8, Sd: 5.07, Range: [0, 22]		
Partner's occupation	Services	4933	40.5%
	Household & domestic	697	5.7%
	Agriculture	3361	27.6%
	Clerical	1752	14.4%
	Prof./Tech./Manag.	497	4.1%
	Did not work	936	7.7%
Mother is currently working	No	9045	74.3%
	Yes	3131	25.7%
Sex of household head	Male	10958	89.8%
	Female	1247	10.2%
			
*Regional characteristics*			
State of residence	29 states of India, see Figure 4a		
Urban/rural location	Urban	4429	36.4%
	Rural	7747	63.6%
			
*Household food competition*			
Number of household members	Mean: 6.68, Median: 6, Sd: 3.16, Range: [2], [35]		
Birth order	Mean: 2.64, Median: 2, Sd: 1.82, Range: [1], [14]		
(including dead children)			
Preceding birth interval [months]	Mean: 26.53, Median: 25, Sd: 25.39, Range: [0, 250]		
Child is twin or multiple birth	No	12037	98.9%
	Yes	139	1.1%
			
*Water, sanitation and hygiene*			
Drinking water in household	Unimproved	2164	17.8%
(according to WHO/UNICEF	Improved	6879	56.5%
classification)	Piped	3133	25.7%
Sanitation facility in household	Unimproved	8345	68.5%
(according to WHO/UNICEF	Improved	3831	31.5%
classification)			
*Indoor air pollution*			
Main cooking fuel	Straw/crop/animal dung	1969	16.2%
	Coal/charcoal/wood	6598	54.2%
	Kerosene	388	3.2%
	Gas/electricity	3221	26.4%
			
*Curative and preventive healthcare*			
Vaccination index	None (0)	1093	9.0%
(Cumulative recommended	Low (1–3)	2106	17.3%
vaccine shots against	Medium (4–6)	2364	19.4%
BCG (1), DPT (3), polio (4)	High (7–9)	6613	54.3%
and measles (1))			
Number of antenatal visits	Mean: 3.91, Median: 3, Sd: 3.44, Range: [0, 26]		
			
*Breastfeeding practices*			
Breastfeeding	No breastfeeding	1578	13.0%
	Breastfeeding + complementary feeding	9450	77.6%
	Exclusive breastfeeding	1148	9.4%
			
*Complementary feeding practices*			
Food diversity	Low (0–2)	7166	58.9%
(Number of food groups	Medium (3–4)	3466	28.5%
consumed during last 24	High (5–8)	1544	12.7%
hours other than breast milk)			
Meal frequency	Low (0–1)	4145	34.0%
(Number of meals consumed	Medium (2–3)	5822	47.8%
during last 24 hours	High (4–9)	2209	18.1%
other than breast milk)			
			
*Micronutrient deficiencies*			
Child ever received iron	No	11464	94.2%
supplements	Yes	712	5.8%
Child ever received vitamin A	No	7724	63.4%
supplements	Yes	4452	36.6%
Iodine-in-salt test result	No iodine	2447	20.1%
(at interview)	Less than 15 parts per million	2775	22.8%
	15 parts per million or more	6954	57.1%

We constructed a three-level variable for breastfeeding and two variables for complementary feeding (**Table 1**). Thereby, food quantity was assessed as meal frequency in the previous 24 hours. Food diversity was measured as the number of food groups a child had consumed in the previous 24 hours, with eight groups defined as food made from grains; food made from roots; food made from beans, peas, lentils, nuts; fruits and vegetables rich in vitamin A; other fruits and vegetables; meat, fish, poultry, eggs; cheese, yoghurt, other milk products; and other food [84]. Grouping of both complementary feeding variables was based on empirical frequencies in our dataset to obtain sufficiently large group sizes.

We defined our study population as the youngest child aged 0–24 months living in each household; not-*de jure* residents were excluded, as several determinants relate to the household environment. Starting from 17039 children, we excluded 2779 children due to missing outcome and 2084 due to missing covariates. The latter were mainly attributable to seven covariates with 50 or more missing values: caste (640 missing values), partner's occupation (212), partner's education (165), drinking water (50), vaccination index (280), number of antenatal visits (153), vitamin A (450), and iodine (118). Our final dataset comprised 12 176 observations; the proportion of missing data was thus about 29%.

### Statistical modelling

We undertook additive quantile regression based on boosting estimation [85], an innovative statistical approach that allows the three underlying research objectives to be investigated simultaneously.

- Quantile regression models quantiles of the outcome as a function of covariates, and therefore enabled us to explore whether covariates exert differential effects across the Z-score distribution, in particular towards the lower tail. In contrast, most analyses of the determinants of undernutrition have used logistic regression models for dichotomized versions of the Z-score (e.g. stunted vs. not stunted) or linear regression models for the continuous Z-score.
- The use of an additive predictor allowed us to explore linear, potentially non-linear, age-varying and spatial effects of the numerous covariates in a flexible way. Additive quantile regression extends conventional linear quantile regression by including flexible functional covariate effects in the predictor while maintaining the assumption of an additive structure. For example, the association between a continuous covariate and the outcome is left unspecified before the analysis and its functional shape is then estimated by, e.g., penalized splines. Most analyses to date have ignored the fact that selected covariates may exert their effects in non-linear and age-varying ways.
- Boosting, a computer-intensive inference method for highly complex models, is currently one of the few possibilities to estimate an additive quantile regression model. As boosting combines parameter estimation and variable selection in one single step, a large number of covariates can be included in the model without requiring subsequent steps of variable selection, as would be the case in classical estimation of quantile or logistic regression. Thereby, boosting estimation enabled us to capture the complex interplay of multiple risk factors in one single model.

We used the following model to assess the impact of stunting determinants on four quantiles of the Z-score:
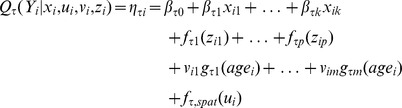



The additive predictor 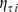 models the conditional quantile function 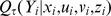 of the outcome 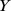 for a fixed quantile parameter 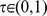 and observation 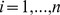. We specified four quantile parameters, namely 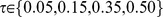. The two values 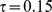 and 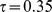 were chosen based on the empirical frequencies for stunting (37%) and severe stunting (17%) in our dataset (**Table 1**), where this choice allows results to be compared across quantile and logistic regression models. 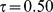 and 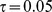 represent the median and an extreme value, respectively.

The flexible additive predictor 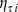 is quantile-specific and comprises linear effects 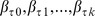 for categorical covariates 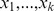, and linear or smooth non-linear effects 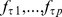 for continuous covariates 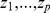. The shapes of the functions 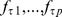 are determined as linear or non-linear in a data-driven way [86] and estimated by the established approach of penalized splines [87]. Also specified are non-linear age-varying effects 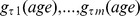 for different levels of the feeding variables 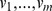; these flexible interaction terms allow meaning and effect of breastfeeding and complementary feeding to vary with age [39]. Further interaction terms were not considered. For the categorical variable 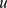, corresponding to 29 Indian states, a smooth spatial function 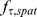 is estimated based on a Gaussian Markov random field [88] to account for spatial autocorrelation and unobserved heterogeneity.

Model estimation was undertaken separately for each 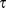 using a component-wise functional gradient descent boosting algorithm [89]. The optimal number of iterations was determined by five-fold cross-validation. The step length was set to 0.2 and each base learner had similar degrees of freedom [90]. Model estimation was repeated on 100 bootstrap samples of the dataset to obtain 95% bootstrap confidence intervals [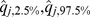] where 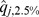 denotes the estimated 2.5 % quantile of 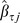, *j = 0,1,…,k*. All analyses were undertaken with the add-on package mboost [91], [92] in the statistical software R [93].

To allow for a comparison with established approaches to investigate child stunting, we also conducted logistic regression analyses for the binary variables stunting and severe stunting. We specified the same flexible predictor and used boosting estimation as described above for quantile regression. This was done to ensure that the conceptual difference between quantile and logistic regression remained as the only explanation for any discrepancies in results.

## Results

**Table 2** shows the results of the 35% and 15% Z-score quantile regression; detailed results of the 50% and 5% Z-score quantile regression are available upon request. (Please note guidance on how statistical significance was assessed in our analysis.) **Table 3** summarizes the results of logistic regression for stunting and severe stunting. All findings on effects of single variables described in text, tables and figures are fully adjusted for other variables.

**Table 2:** Estimated effects and 95% bootstrap confidence intervals for boosting quantile regression models for the 35% Z-score quantile (columns in grey) and the 15% Z-score quantile; see Figures 2 and 3 for detailed results of continuous covariates. 1,2

Variable	Values/Description	Quantile regression for 35% Z-score quantile		Quantile regression for 15% Z-score quantile	
		β_0.35_	95% CI(β_0.35_)	β_0.15_	95% CI(β_0.15_)
*Non-modifiable factors*					
Child age [months]		**∼ Linear, negative**		**∼ Linear, negative**	
Child sex	Male	–	–	–	–
	Female	**0.166**	**[0.103, 0.234]**	**0.209**	**[0.130, 0.285]**
*Maternal characteristics*					
Maternal age [years]		**Non-linear, inverse U**		**Non-linear, inverse U**	
Maternal BMI [kg/m^2^]		**Non-linear, positive**		**Non-linear, positive**	
*Household characteristics*					
Household wealth	Poorest	–	–	–	–
	Poorer	0.025	[−0.077, 0.110]	0.035	[−0.041, 0.129]
	Middle	0.058	[−0.014, 0.161]	0.001	[−0.067, 0.079]
	Richer	0.089	[−0.016, 0.205]	0.075	[−0.014, 0.207}
	Richest	**0.224**	**[0.069, 0.383]**	**0.214**	**[0.060, 0.367]**
Religion of household head	Hindu	–	–	–	–
	Muslim	0.003	[−0.064, 0.086]	0.003	[−0.075, 0.101]
	Christian	0.034	[−0.023, 0.139]	0.089	[−0.001, 0.222]
	Sikh	0.021	[−0.009, 0.116]	0.068	[−0.001, 0.180]
	(Neo-)Buddhist	0.000	[−0.032, 0.034]	−0.006	[−0.085, 0.066]
	Other	−0.006	[−0.064, 0.028]	−0.030	[−0.132, 0.028]
Caste/tribe of household head	Scheduled caste	–	–	–	–
	Scheduled tribe	**0.088**	**[0.005, 0.224]**	0.037	[−0.060, 0.156]
	Other backward class	**0.112**	**[0.034, 0.214]**	**0.115**	**[0.011, 0.213]**
	None of them	**0.165**	**[0.062, 0.294]**	**0.167**	**[0.049, 0.302]**
Maternal education [years]		**∼ Linear, positive**		**∼ Linear, positive**	
Partner's education [years]		**∼ Linear, positive**		**∼ Linear, positive**	
Partner's occupation	Services	–	–	–	–
	Household & domestic	0.035	[−0.021, 0.132]	0.055	[−0.002, 0.179]
	Agriculture	0.028	[−0.031, 0.104]	0.042	[−0.015, 0.136]
	Clerical	0.013	[−0.039, 0.079]	0.005	[−0.059, 0.077]
	Prof./Tech./Manag.	0.037	[−0.015, 0.132]	−0.011	[−0.105, 0.069]
	Did not work	0.009	[−0.062, 0.082]	−0.009	[−0.092, 0.049]
Mother is currently working	No	–	–	–	–
	Yes	**−0.078**	**[−0.152, −0.001]**	−0.044	[−0.122, 0.018]
Sex of household head	Male	–	–	–	–
	Female	0.029	[−0.033, 0.124]	0.023	[−0.037, 0.113]
*Regional characteristics*					
State of residence		**Spatial, see ** **Figure 4b**		**Spatial**	
Urban/rural location	Urban	–	–	–	–
	Rural	−0.002	[−0.074, 0.071]	0.025	[−0.076, 0.113]
*Household food competition*					
Number of household members		Non-linear, inverse U		Non-linear, inverse U	
Birth order		**Non-linear, negative**		**Non-linear, negative**	
Preceding birth interval [months]		**Non-linear, positive**		Non-linear, positive	
Child is twin or multiple birth	No	–	–	–	–
	Yes	**−0.866**	**[−1.107, −0.456]**	**−0.890**	**[−1.173, −0.497]**
*Water, sanitation and hygiene*					
Drinking water in household	Unimproved	–	–	–	–
	Improved	−0.026	[−0.093, 0.015]	−0.004	[−0.056, 0.051]
	Piped	−0.007	[−0.078, 0.026]	0.003	[−0.036, 0.043]
Sanitation facility in household	Unimproved	–	–	–	–
	Improved	**0.092**	**[0.041, 0.159]**	**0.114**	**[0.031, 0.227]**
					
*Indoor air pollution*					
Main cooking fuel	Straw/crop/animal dung	–	–	–	–
	Coal/charcoal/wood	−0.040	[−0.090, 0.015]	−0.031	[−0.105, 0.027]
	Kerosene	−0.020	[−0.081, 0.007]	−0.056	[−0.164, −0.001]
	Gas/electricity	0.055	[−0.009, 0.170]	**0.076**	**[0.001, 0.179]**
*Curative and preventive healthcare*					
Vaccination index	None (0)	–	–	–	–
	Low (1–3)	−0.015	[−0.079, 0.033]	0.010	[−0.053, 0.073]
	Medium (4–6)	−0.026	[−0.081, 0.043]	−0.031	[−0.100, 0.033]
	High (7–9)	**0.062**	**[0.004, 0.137]**	**0.080**	**[0.007, 0.175]**
Number of antenatal visits		**Non-linear, inverse U**		**Non-linear, inverse U**	
					
*Breastfeeding practices*					
Breastfeeding	No breastfeeding	–	–	–	–
	Breastfeeding + complementary feeding	**Non-linear, negative by age**		**Non-linear, negative by age**	
	Exclusive breastfeeding	**Non-linear, negative by age**		**Non-linear, negative by age**	
*Complementary feeding practices*					
Food diversity	Low (0–2)	–	–	–	–
	Medium (3–4)	Constant, positive by age		Constant, positive by age	
	High (5–8)	**∼ Linear, positive by age**		**∼ Linear, positive by age**	
Meal frequency	Low (0–1)	–	–	–	–
	Medium (2–3)	Constant, zero by age		Constant, zero by age	
	High (4–9)	∼ Linear, positive by age		∼ Linear, positive by age	
*Micronutrient deficiencies*					
Child ever received iron	No	–	–	–	–
supplements	Yes	−0.025	[−0.123, 0.045]	−0.049	[−0.168, 0.035]
Child ever received vitamin A	No	–	–	–	–
supplements	Yes	**0.076**	**[0.005, 0.140]**	0.046	[0.000, 0.121]
Iodine-in-salt test result	No iodine	–	–	–	–
	Less than 15 parts per million	−0.035	[−0.093, 0.058]	−0.063	[−0.134, 0.014]
	15 parts per million or more	**0.097**	**[0.037, 0.164]**	**0.095**	**[0.036, 0.162]**

1 Significant effects are shown in bold. An effect of a categorical covariate is rated as significant if the corresponding 95% bootstrap confidence interval does not contain zero. An effect of a continuous covariate is rated as significant if the effects from all 100 bootstrap samples are estimated to be below/above zero for at least one interval within the covariate range.

2 The effects of categorical covariates can be interpreted as their effect on the respective Z-score quantile relative to the reference category. For example, the 35% quantile of the Z-score for girls is significantly increased by 0.166 compared to the 35% quantile of boys, given all other covariates are similar.

**Table 3:** Estimated effects and 95% bootstrap confidence intervals for boosting logistic regression models for the binary variables stunting and severe stunting.^1,2,3^

Variable	Values/Description	Logistic regression for stunting		Logistic regression for severe stunting	
		β_stunted_	95% CI(β_stunted_)	β_sevSt_	95% CI(β_sevSt_)
*Non-modifiable factors*					
Child age [months]		**∼ Linear, positive**		**∼ Linear, positive**	
Child sex	Male	–	–	–	–
	Female	**−0.080**	**[−0.123, −0.037]**	**−0.120**	**[−0.171, −0.068]**
*Maternal characteristics*					
Maternal age [years]		**Non-linear, U-shape**		**Non-linear, U-shape**	
Maternal BMI [kg/m^2^]		**Non-linear, U-shape**		**Non-linear, negative**	
*Household characteristics*					
Household wealth	Poorest	–	–	–	–
	Poorer	0.007	[−0.045, 0.063]	−0.044	[−0.104, 0.026]
	Middle	−0.011	[−0.058, 0.031]	−0.056	[−0.129, −0.002]
	Richer	−0.041	[−0.115, 0.019]	**−0.119**	**[−0.235, −0.030]**
	Richest	**−0.130**	**[−0.244, −0.027]**	**−0.221**	**[−0.353, −0.085]**
Religion of household head	Hindu	–	–	–	–
	Muslim	−0.045	[−0.114, 0.010]	−0.004	[−0.058, 0.059]
	Christian	−0.037	[−0.119, 0.038]	−0.017	[−0.087, 0.033]
	Sikh	−0.046	[−0.124, 0.004]	−0.013	[−0.060, 0.014]
	(Neo-)Buddhist	−0.023	[−0.126, 0.033]	−0.016	[−0.093, 0.020]
	Other	0.041	[−0.002, 0.118]	0.026	[−0.014, 0.103]
Caste/tribe of household head	Scheduled caste	–	–	–	–
	Scheduled tribe	−0.030	[−0.100, 0.021]	−0.038	[−0.120, 0.026]
	Other backward class	**−0.066**	**[−0.126, −0.009]**	**−0.078**	**[−0.132, −0.025]**
	None of them	**−0.112**	**[−0.188, −0.047]**	**−0.134**	**[−0.224, −0.064]**
Maternal education [years]		**∼ Linear, negative**		**∼ Linear, negative**	
Partner's education [years]		**∼ Linear, negative**		**∼ Linear, negative**	
Partner's occupation	Services	–	–	–	–
	Household & domestic	−0.030	[−0.090, 0.010]	−0.056	[−0.152, 0.008]
	Agriculture	−0.006	[−0.042, 0.032]	−0.055	[−0.111, −0.012]
	Clerical	−0.011	[−0.047, 0.038]	−0.030	[−0.093, 0.026]
	Prof./Tech./Manag.	−0.014	[−0.064, 0.032]	0.016	[−0.030, 0.090]
	Did not work	0.001	[−0.045, 0.049]	0.015	[−0.026, 0.085]
Mother is currently working	No	–	–	–	–
	Yes	0.043	[0.000, 0.086]	0.040	[0.000, 0.093]
Sex of household head	Male	–	–	–	–
	Female	−0.023	[−0.081, 0.003]	−0.006	[−0.067, 0.036]
*Regional characteristics*					
State of residence		**Spatial**		**Spatial**	
Urban/rural location	Urban	–	–	–	–
	Rural	−0.045	[−0.093, 0.000]	−0.021	[−0.071, 0.000]
*Household food competition*					
Number of household members		Non-linear, U shape		Non-linear, U shape	
Birth order		Non-linear, positive		∼ Linear, positive	
Preceding birth interval [months]		Non-linear, negative		Non-linear, negative	
Child is twin or multiple birth	No	–	–	–	–
	Yes	**0.420**	**[0.251, 0.579]**	**0.566**	**[0.385, 0.750]**
*Water, sanitation and hygiene*					
Drinking water in household	Unimproved	–	–	–	–
	Improved	0.019	[−0.005, 0.063]	−0.005	[−0.045, 0.029]
	Piped	0.010	[−0.025, 0.068]	−0.006	[−0.053, 0.019]
Sanitation facility in household	Unimproved	–	–	–	–
	Improved	**−0.057**	**[−0.111, −0.011]**	**−0.049**	**[−0.112, −0.001]**
*Indoor air pollution*					
Main cooking fuel	Straw/crop/animal dung	–	–	–	–
	Coal/charcoal/wood	0.014	[−0.018, 0.044]	0.005	[−0.036, 0.055]
	Kerosene	0.018	[−0.028, 0.058]	**0.124**	**[0.019, 0.238]**
	Gas/electricity	**−0.088**	**[−0.168, −0.015]**	−0.065	[−0.145, 0.001]
*Curative and preventive healthcare*					
Vaccination index	None (0)	–	–	–	–
	Low (1–3)	−0.005	[−0.074, 0.038]	−0.004	[−0.076, 0.037]
	Medium (4–6)	−0.004	[−0.086, 0.044]	0.006	[−0.099, 0.052]
	High (7–9)	**−0.072**	**[−0.151, −0.013]**	**−0.059**	**[−0.152, −0.005]**
Number of antenatal visits		∼ Linear, negative		Non-linear, U shape	
*Breastfeeding practices*					
Breastfeeding	No breastfeeding	–	–	–	–
	Breastfeeding + complementary feeding	**Non-linear, positive by age**		**Non-linear, positive by age**	
	Exclusive breastfeeding	∼ Linear, positive by age		∼ Linear, positive by age	
*Complementary feeding practices*					
Food diversity	Low (0–2)	–	–	–	–
	Medium (3–4)	Constant, zero by age		Constant, negative by age	
	High (5–8)	∼ **Linear, negative by age**		∼ **Linear, negative by age**	
Meal frequency	Low (0–1)	–	–	–	–
	Medium (2–3)	Constant, zero by age		Constant, zero by age	
	High (4–9)	Constant, zero by age		Constant, zero by age	
*Micronutrient deficiencies*					
Child ever received iron	No	–	–	–	–
supplements	Yes	0.022	[−0.016, 0.089]	0.030	[−0.007, 0.138]
Child ever received vitamin A	No	–	–	–	–
supplements	Yes	−0.036	[−0.077, 0.000]	−0.020	[−0.070, 0.000]
Iodine-in-salt test result	No iodine	–	–	–	–
	Less than 15 parts per million	0.011	[−0.043, 0.044]	0.025	[−0.013, 0.058]
	15 parts per million or more	**−0.056**	**[−0.107, −0.020]**	**−0.066**	**[−0.118, −0.022]**

**Figure 2:**
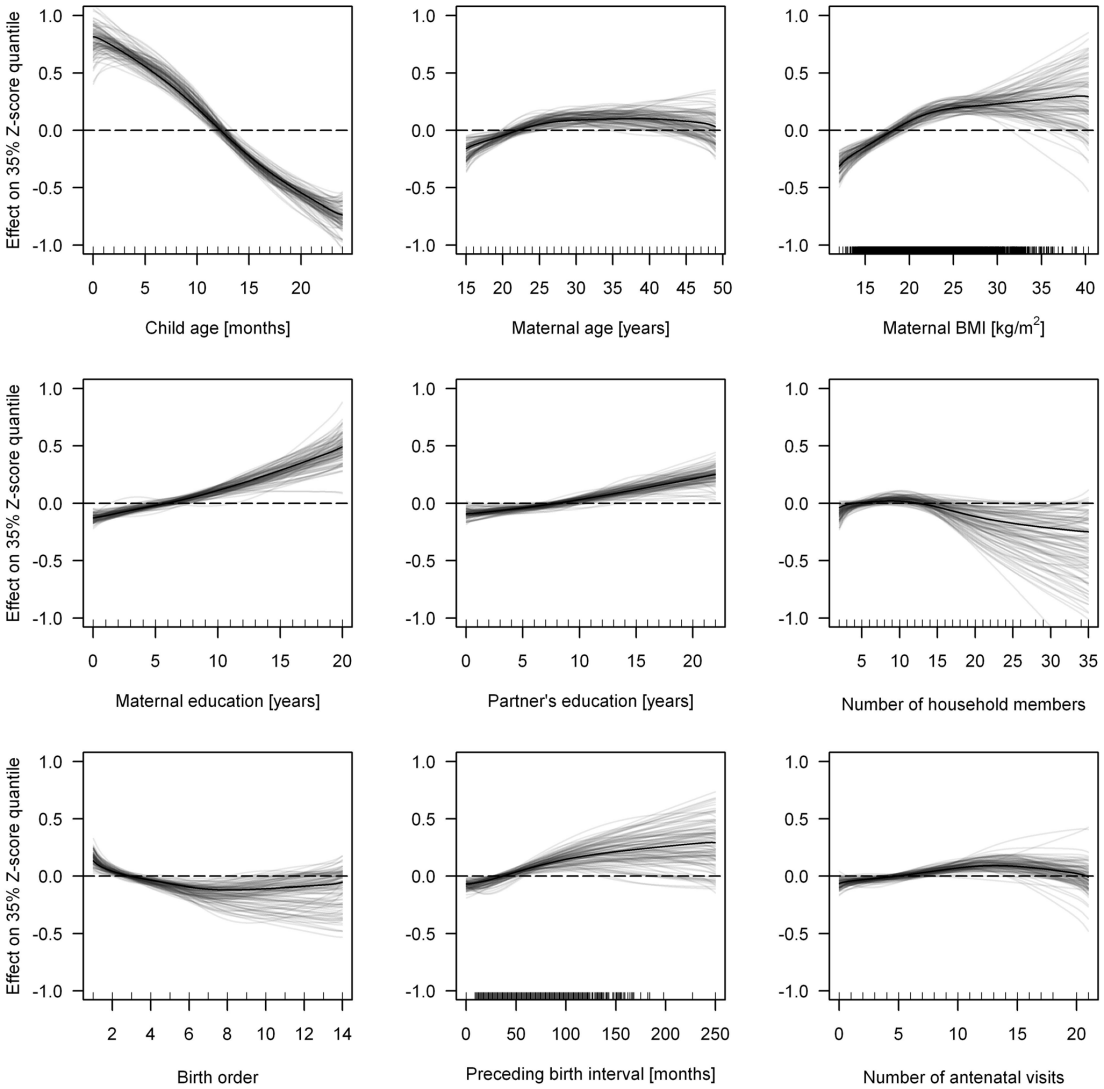
Linear or smooth non-linear effects of continuous covariates from 35% quantile regression for the full model (black line) and 100 bootstrap iterations (grey lines).

1 Significant effects are shown in bold; please see Figure 2, footnote 1, on how statistical signifance is assessed.

2 The effect of a covariate in logistic regression relates to the log-odds ratio for being stunted or severely stunted (in contrast to quantile regression where an effect relates to the respective quantile of the Z-score). For example, the log-odds ratio for being stunted for girls is −0.080 smaller compared to boys, given all other covariates are similar.

3 Absolute values of effects from Table 3 cannot be compared to those from Table 2, but reversed effect signs indicate concordant results from quantile and logistic regression.

Here, we focus on the results of the 35% Z-score quantile regression, which corresponds to the empirical frequency for stunting (37%) in our dataset and therefore allows the results to be compared with those of logistic regression for being stunted. Importantly, except for the indoor air pollution group, at least one variable in each of the eleven assessed groups of determinants shows a statistically significant association with the 35% Z-score quantile. With respect to our research objectives, this suggests that an integrated analysis of the multiple immediate, intermediate and underlying determinants of stunting is merited.

**Table 2** shows the effects for categorical covariates and their 95% bootstrap confidence intervals, and summarizes the shape of the function for continuous variables. The following categorical covariates have at least one significant category compared with the reference category: child sex, household wealth, caste of household head, mother is currently working, child is twin, sanitation facility, vaccination index, vitamin A and iodine. For example, the 35% Z-score quantile for children from the richest households is significantly increased by 0.224 [0.069, 0.383] compared to children from the poorest households. Being a twin has a very large negative effect of −0.866 [−1.107, −0.456].

**Figure 2** shows the effects of continuous covariates estimated from the full model and 100 bootstrap iterations. With the exception of number of household members, all continuous variables show significant non-zero effects in all bootstrap samples. Child age shows the largest absolute effect size: the 35% Z-score quantile decreases by almost two units from birth until the age of 24 months.

Non-linear functions are estimated for maternal age and BMI, birth order, preceding birth interval and the number of antenatal visits (**Figure 2**). The effect of maternal age increases linearly until 30 years, then remains constant and gradually decreases from 45 years. Height-for-age increases monotonically with greater maternal BMI, with the slope reducing at 25 kg/m^2^. Birth order shows a linearly decreasing effect until the 6^th^ child and then remains constant, while lengthening the interval between births is associated with increased height-for-age up until 100 months. The effect of the number of antenatal visits has a slight inverse U-shape, where low and high numbers of antenatal visits are associated with smaller 35% quantiles than medium numbers (8–15 visits). With respect to our research objectives, the observed non-linear functions emphasize that selected determinants of stunting exert their effects in non-linear ways.

**Figure 3:**
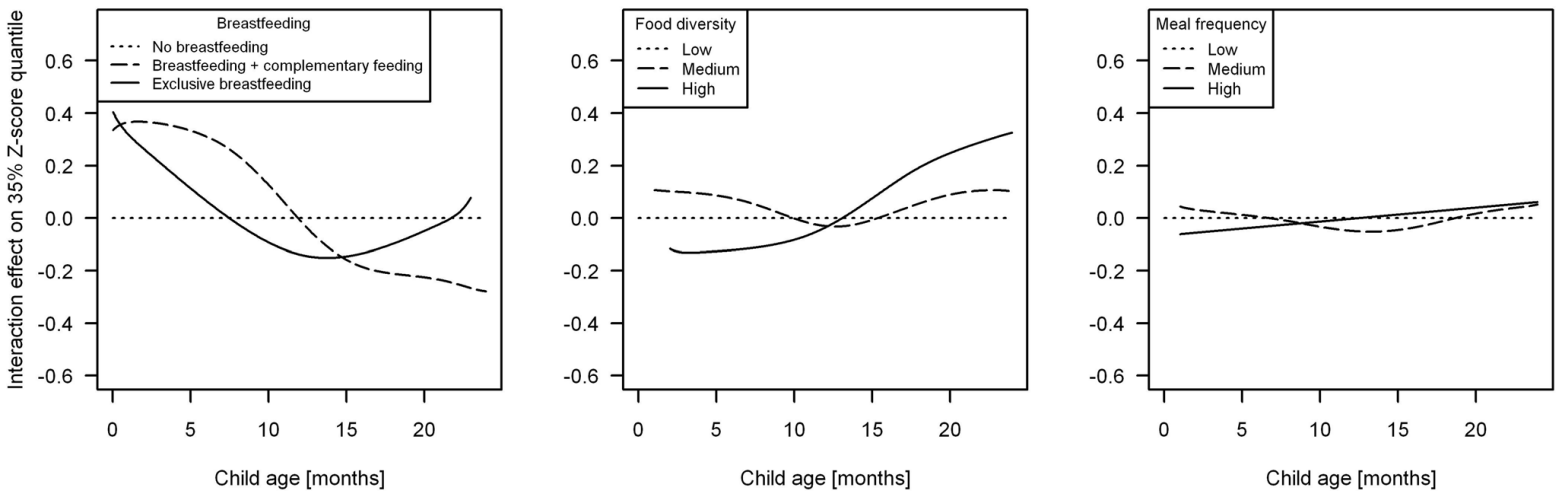
Non-linear age-varying effects of feeding variables estimated by 35% quantile regression (full model). The dotted horizontal line at zero represents the respective reference category.

**Figure 3** depicts the age-varying effects of feeding variables. The effect of breastfeeding on the 35% Z-score quantile clearly varies with age: any breastfeeding compared to no breastfeeding exerts a positive effect until 9 months followed by a negative effect beginning at 12 months; the increasing effect of exclusive breastfeeding after 14 months is based on small numbers and shows large variation. Compared to low food diversity, high diversity exerts a significantly negative effect until the age of 12 months, and a significantly positive effect thereafter; medium food diversity does not differ significantly from the reference category. No significant differences in relation to meal frequency are observed.

**Figure 4:**
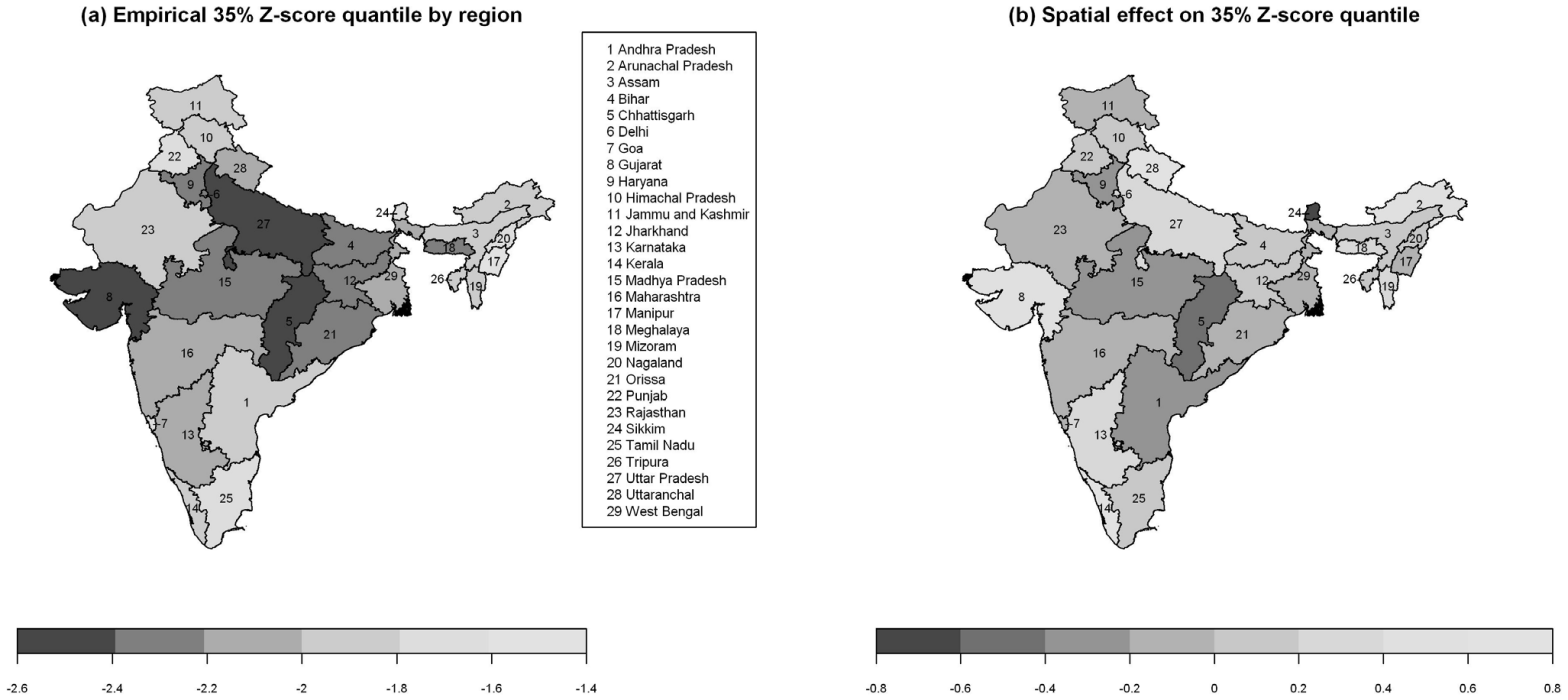
Empirical 35% Z-score quantile of child stunting by region (a), and smooth spatial effect estimated by 35% quantile regression for the 29 Indian states (b).

**Figure 4** displays the empirically observed 35% Z-score quantiles for 29 Indian states, showing stark differences in stunting (**Figure 4a**), and the estimated spatial effect on the 35% Z-score quantile for state of residence (**Figure 4b**). Less pronounced differences in **Figure 4b** compared to **Figure 4a** imply that model covariates offer a partial explanation for regional differences.

There are no fundamental differences between the results for the 35% Z-score quantile and those for other quantiles (see **Table 2** for 15% Z-score quantile). The majority of categorical, continuous and age-varying variables described above also show significant effects of the same direction and of a similar size for the 15% and 50% Z-score quantiles; for the extreme 5% Z-score quantile, some of these variables are no longer significant. Two categorical variables, however, only show statistical significance in analyses of one quantile: mother is working (35% Z-score quantile) and main cooking fuel (15% Z-score quantile). The above described non-linear effects are very similar across all quantiles, even for the 5% Z-score quantile. The only difference with regard to linearity vs. non-linearity is detected for maternal education (linear for 15% and 35%, non-linear for 5% and 50%).

Likewise, the differences between the results for quantile and logistic regression models are limited (**Table 3** **;** please note guidance on interpretation of effect estimates in logistic vs. quantile regression). Most statistically significant variables across the four quantiles also show significance in logistic regression analyses. Exclusive breastfeeding, birth order, number of antenatal visits and vitamin A, however, show no effects on stunting and severe stunting. In contrast, main cooking fuel is statistically significant in both logistic regression models.

With respect to our research objectives, the mostly consistent results across different Z-score quantiles and modelling approaches suggest that risk factors do not appear to show differential effects across the height-for-age distribution.

## Discussion

### Key findings

We employed an evidence-based, systematic approach to identify all likely determinants of child stunting and to capture the interconnectedness between multiple risk factors within the system. For each of the eleven groups of determinants we conceptualized in **Figure 1** and were able to populate with variables from the Indian NFHS, we found at least one variable with a statistically significant effect in all quantile and logistic regression models – except for the indoor air pollution group, which only showed a significant effect in three out of six regression models. This emphasizes the broad range of causes of child stunting, encompassing more distal maternal, household socio-economic and regional characteristics as well as more proximate environmental, nutrition, infection-related and healthcare-related determinants. It suggests many potential entry-points for intervention and offers some insights regarding high-risk groups. Yet, our analysis also implies that a less comprehensive approach may overlook key determinants of stunting, potentially resulting in incorrect effect estimates in analyses of risk factors or leading to interventions that do not sufficiently take context into account.

Looking more closely within groups of determinants, our analysis confirms the importance of child age and sex as non-modifiable determinants and highlights household wealth, greater maternal education and greater maternal BMI as major protective factors, given the large and statistically significant effects of these variables. The findings regarding household characteristics, such as household wealth and maternal education [62]–[65], and maternal nutrition status [70]–[72] mirror those in the literature. Our research also draws attention to twins as a potentially overlooked risk group [75]; the very large significantly negative effect is remarkable, as only 1% of children in the NFHS dataset are twins or multiple births. On the other hand, none of the models detected statistically significant effects of religion of household head, partner's occupation, sex of household head, urban/rural location, number of household members, drinking water, meal frequency by age, or iron supplementation, which contrasts with previous reports [30], [33], [34], [41], [42], [43], [46], [47], [48], [78]. This may be due to the poor quality of the proxy measures we employed or differences in the population distribution of variables [94]. Most importantly, it may reflect the fact that in a more comprehensive model, the effect of some variables is captured by other related variables.

Statistical modelling was realized by additive quantile regression to explore whether differential effects emerge across the height-for-age distribution and to investigate the presence of non-linear effects. The results across the four quantile and two logistic regression analyses were largely comparable, suggesting that the impact of most of the variables on lower tails of the height-for-age distribution does not differ from their impact on the population mean. We attribute this lack of differential effects to the symmetric shape of the height-for-age Z-score distribution which is independent of covariates. Therefore, using the more established logistic regression instead of quantile regression is likely to be appropriate in most analyses of the determinants of child stunting. Importantly, this research has demonstrated that maternal age, maternal BMI, and birth order exert their effect in a non-linear way; for maternal age and BMI these findings are in line with previous results [23]. Thus, assuming linearity in statistical modelling could lead to incorrect conclusions. To avoid inappropriate oversimplification, we propose that logistic or quantile regression models of stunting determinants should take a systems-based approach to analysis and explicitly consider potential non-linear effects.

### Strengths and limitations of this study

#### Data quality

An inherent limitation of cross-sectional data is their snapshot nature, which makes establishing a temporal sequence of events and drawing causal inferences impossible. Moreover, while the NFHS includes suitable variables for most determinants of stunting, we could not model the impact of immediate determinants, were unable to populate the groups of determinants chronic diseases and recurrent infections and could only partially assess micronutrient deficiencies, healthcare, maternal or regional characteristics. Similarly, some of the proxies we used in our analysis may not provide an accurate estimate of the underlying concept of interest (e.g. type of cooking fuel as a proxy for indoor air pollution). Consequently, effect sizes for individual variables should be interpreted with caution. Even though the NFHS is considered a high-quality dataset, the logical consequence of assessing a large number of potential determinants was a high proportion of missing data (about 29%). Large numbers of missing values in selected variables, in particular in the outcome of interest, may have introduced selection bias. Indeed, compared to children with Z-score information, children for whom the outcome variable was missing were more likely to be younger and a twin (factors that increase stunting risk), as well as more likely to be born to mothers with greater maternal BMI and to live in wealthier and urban households (factors that decrease stunting risk). All differences were small, and are likely to increase uncertainty in effect estimates for these variables, thereby biasing results towards the null. Nevertheless the large-scale, standardized and nationally representative nature of the NFHS, a response rate of eligible women of 94.5% [84] and coverage of a broad range of health risks makes this data source ideally-suited for a comprehensive analysis of stunting determinants. Also, a recent methodological study suggests that cross-sectional studies can yield reliable estimates for risk factors that vary more across space at a fixed point in time than at a fixed location across different points in time [95].

#### Evidence-based approach

Based on earlier work in this field [25], a priori reasoning and extensive searches of the literature, we derived a schematic diagram of the multiple determinants of stunting. One limitation of this diagram is that it does not explicitly cover macro-level factors, such as good governance, peace and stability or climate change [18], [79], factors that are likely to be relatively constant within a given country but that may be major underlying causes for cross-country differences in child undernutrition [94]. In addition, we neither examined the hierarchical structure contained within this diagram nor the pathways and relationships between individual determinants. Nevertheless, we believe that our approach to identifying all likely determinants of stunting and to populating as many of these as possible using an existing dataset is novel and takes up recent calls to incorporate systems thinking in epidemiology [19]–[22], [96].

#### Statistical methods

Statistical modelling was realized by the innovative statistical approach of additive quantile regression based on boosting estimation since this method allowed us to simultaneously investigate our three research objectives. As extension of classical linear quantile regression, the flexible predictor of additive quantile regression enables potentially non-linear functional shapes of continuous covariates to be determined in a data-driven way and to account for spatial autocorrelation by including smooth spatial effects. Boosting combines parameter estimation and variable selection in one single estimation step, making it ideally suited to models with a large number of covariates, since subsequent steps of variable selection are not required. An inherent limitation of boosting is the lack of standard errors which makes the use of re-sampling methods, such as bootstrap, necessary to assess the variability of effect estimates. As a consequence, with boosting statistical significance cannot be assessed in a traditional way (i.e. based on test statistics with well-known distributions). In our analysis, we instead derived statistical significance from the bootstrap results. For a categorical covariate, for example, significance was defined as having at least one significant category compared with the reference category; and overall tests could not be conducted. A strength of boosting estimation is that it can be applied independently of the scale of the outcome and of the corresponding regression model, i.e., linear, quantile, or binary regression, as was demonstrated in our logistic regression analysis. On the other hand, an important limitation of our statistical modelling approach is that it does not explicitly account for the hierarchy implied by the conceptual diagram.

### Implications for research and practice

Clearly, this research is located at the very beginning of a lengthy, cyclical process to develop and implement complex interventions, which comprises formative research and piloting as well as randomized controlled trials and implementation research [97]; and some of the insights might be specific for the Indian sub-continent. Do the insights gained impact in any way on how we might design and implement interventions more successfully?

The multi-factorial nature of child stunting offers many entry-points for technical and policy solutions and suggests that, ultimately, the impact of any intervention is influenced by the combined effects of all of these groups of determinants within the system. If we fully accept this notion, the finding that many single interventions show rather limited health impact is not surprising. Indeed, initial findings from the Millennium Villages project suggest that a combination of nutrition-specific, health-based approaches with food system- and livelihood-based interventions can achieve substantial reductions in childhood stunting [98], although the approach to analysis likely overstates the impact of the intervention [99]. Embracing systems thinking, it also becomes clear that the design and implementation of interventions must not take place out of context and that “context” goes beyond a broad distinction between food-secure and food-insecure populations [17], [100]. A range of socio-economic, cultural and climatic factors at household, community and national levels impacts the choice of universal *versus* targeted approaches [101], [102], [60] and other specific aspects related to the design and delivery of intervention packages.

Revisiting the determinants of child stunting is timely in view of recent calls to set up a national nutrition strategy for India, which would combine food and nutrition programmes with broad investments in health, sanitation, agriculture and women's status [101], emphasizing multi-sectoral coordination to assure that “every link in the chain of malnutrition (is) considered” [102]. It is also relevant with respect to the global hunger summit hosted during the London Olympics 2012 and commitments to invest in a range of measures to reduce child malnutrition prior to the Rio Olympics in 2016. We hope that the insights offered here will add food for thought in relation to how these pledges are put into practice.
